# Mental health provider perspectives of the COVID-19 pandemic impact on service delivery: a focus on challenges in remote engagement, suicide risk assessment, and treatment of psychosis

**DOI:** 10.1186/s12913-022-08106-y

**Published:** 2022-05-31

**Authors:** Lindsay A. Bornheimer, Juliann Li Verdugo, Joshua Holzworth, Fonda N. Smith, Joseph A. Himle

**Affiliations:** 1 University of Michigan, School of Social Work, Ann Arbor, MI USA; 2 University of Michigan, Department of Psychiatry, Ann Arbor, MI USA

**Keywords:** Mental health, Service delivery, Suicide, Risk assessment, Community mental health, Psychosis

## Abstract

**Background:**

The COVID-19 pandemic has been impacting the need, utilization, and delivery of mental health services with greater challenges being faced by clients and providers. With many clients facing reduced access to services and social isolation, a focus on suicide risk assessment and prevention is critical. Concern is particularly increased for clients with schizophrenia spectrum disorders given data show suicide rates are disproportionately high for those with psychosis in comparison to the general population. Provider perspectives of challenges in service delivery are needed to inform efforts to improve access, feasibility, and quality of mental health care throughout the evolving pandemic. This study explored mental health provider perspectives of client challenges in service utilization and provider challenges in service delivery, including remote engagement, suicide risk assessment, and treatment of psychosis.

**Methods:**

Data were collected from social work mental health providers (*n* = 12) in United States community mental health setting. Providers consented to participate and responded to questions about service delivery experiences in late 2020 and in relation to COVID-19. Demographic and practice-related provider data were explored descriptively using SPSS and qualitative data using open coding and grounded theory methods in Dedoose.

**Results:**

Among the 9 providers who engaged in remote service delivery, 7 (77.8%) experienced challenges in remote engagement with clients and 8 (88.9%) experienced challenges in treatment of psychosis. Among the 7 providers who engaged in remote suicide assessment, 4(57%) experienced challenges. Qualitative themes emerged including logistic (e.g., technology access and use), engagement (e.g., virtual rapport-building and limited remote services), and clinical (e.g., difficulty assessing suicide risk, internal stimuli, abnormal involuntary movement, and affect) challenges in service delivery.

**Conclusions:**

Provider perspectives are essential to inform efforts to build resources and problem-solve challenges and barriers that both providers and clients face throughout various shifts in mental health service delivery. Findings emphasize the need to troubleshoot client access to technology, bolster support for providers to prevent burnout, and greater provider training to improve skills in remote engagement, assessment, and treatment, particularly in relation to psychosis and suicide prevention. Study implications are not only critical for the evolving COVID-19 pandemic, but also in preparation for ongoing shifts in service delivery as technology evolves.

## Introduction

The COVID-19 pandemic has led to substantial psychological impact in the United States and worldwide [1–3]. For many individuals, mental health needs have been rising throughout the pandemic’s progression [4], with adjustment-related impacts of quarantine, health anxiety, economic and financial stressors, social isolation, and more [5, 6]. Aligning with preexisting epidemic and outbreak-related studies [7–9], the COVID-19 pandemic has prompted greater anxiety and depression [10], social isolation [11], and risk for suicide [12]. While the pandemic’s impact on mental health has been increasingly investigated, greater understandings of its impact on mental health services and delivery are needed from the perspective of mental health providers. Furthermore, and importantly, less is known to date about challenges providers community mental health (CMH) face in remote assessment and treatment of psychosis and suicide risk in the United States.

Suicide is a critical public health problem and leading cause of preventable death among individuals with schizophrenia spectrum and other psychotic disorders [13], Bornheimer, 2020) [14]. Data estimate the risk for suicide among individuals with schizophrenia spectrum disorders is over eight times greater than the general population [15]. It is predicted that short- and long-term impact of the COVID-19 pandemic may disproportionately affect individuals with psychosis [16] and given the potential for worsening mental health symptoms [6], there are rising concerns of suicide risk within this population [17]. As a result, it is essential that mental health providers gain skills to effectively conduct comprehensive suicide-risk assessments, formulate levels of risk, and deliver treatments and services to reduce risk and prevent death. Furthermore, CMH settings are well-positioned to engage in suicide prevention efforts as they are among the largest providers of outpatient behavioral health in the United States, and particularly so for clients with serious mental illness and psychosis [18].

Service delivery has increasingly shifted to telehealth and virtual formats throughout the COVID-19 pandemic with notable impact on individuals seeking care, mental health providers adjusting to remote service delivery, and an increased need for mental health services [19, 20]. In the United States in particular, the demand for mental health services have remarkably increased [21]. Across the globe and within numerous individual contexts, there are varying perceptions about and experiences with virtual healthcare services among providers and clients. For some clients, virtual services increase accessibility to care, and for others, technology barriers have made care more inaccessible when face-to-face services are not an option [22]. Among providers, there is a similar spread of virtual services being experienced as acceptable and effective in practice, yet also challenging given technology access and clinical barriers (e.g., not being able to observe certain nonverbal cues and less privacy) that may arise [1, 23, 24]. One qualitative study exploring healthcare provider experiences during the pandemic yielded themes of a prevailing sense of helplessness, overwhelming workloads for providers, and increased mental health decline among clients [25]. It is evident that providers have been impacted throughout the onset and progression of the COVID-19 pandemic. While the pandemic’s overall impact on mental health services is increasingly investigated, greater understandings are needed from provider perspectives regarding the impact on service delivery in United States CMH settings, such as perceived challenges of clients receiving treatment and provider challenges in workload, engagement, assessment, and treatment. The CMH context in the United States is particularly important, given many clients engaging in community-based mental health services reside in low-income, underserved, or rural areas with less access to technology [22]. Additionally, there are gaps in knowledge about the pandemic’s impact on provider assessment and treatment of both symptoms of psychosis and suicide risk. In particular, the remote nature of service delivery likely poses complexities in client engagement, assessment of mental status and symptoms, and delivery of behavioral interventions.

Given ongoing changes in mental health service delivery and increased need for care, emerging research from the perspective of providers in CMH is particularly vital to inform clinical practice and future research aiming to disseminate mental health services in mental health service systems. The current study explored provider perspectives of mental health services and delivery challenges in relation to the COVID-19 pandemic with specific focus on providing services to individuals with psychosis symptoms and at risk for suicide.

## Methods

Data were collected as part of a National Institute of Mental Health (NIMH)-funded study (R34MH123609; PI: Bornheimer) focused on suicide prevention among adults with psychosis in a CMH setting. As a first step in the study, survey data were collected in the fall of 2020 to explore the impact of the COVID-19 pandemic on providers delivering mental health services. This manuscript presents our pandemic-related demographic and qualitative data among social work mental health providers in a CMH setting and aligns with the consolidated criteria for reporting qualitative studies (COREQ; [26].

### Setting, Participants, and Procedures

A total of 12 mental health providers in a midwestern CMH setting of the United States participated in this study. Using purposive sampling methods, providers were recruited through informational presentations given in virtual provider staff meetings. This CMH setting provides mental health services to adults with severe mental illness and developmental disability, offering a breadth of programs including crisis residential services, case management, outpatient mental health, medication management, assertive community treatment (ACT), and more. Prior to the pandemic, the majority of services in this CMH setting were delivered in-person (e.g., individual and group psychotherapy, psychiatric evaluations, medication management, and case management), with few services being delivered outside of CMH facilities (e.g., ACT for clients with serious and persistent mental illness involving multidisciplinary care in their home and community). The range of attendance in the CMH facility ranged by service type, with some clients attending multiple times a week for therapeutic services, many attending once or twice a month for case management and medication management, and a smaller subset attending every few months. Once the pandemic began to impact this region of the United States in early 2020, many services became limited for CMH clients (e.g., case management, medication management, and therapy sessions occurred less often than typical) and some services were temporarily discontinued (e.g., group psychotherapy). Importantly, and uncommon in CMH prior to the pandemic, teletherapy and virtual service delivery became a standard approach to care when local lockdowns occurred, and safety protocols were put in place with less in-person access to the CMH facility to prevent the spread of COVID-19. ACT continued in the community with home visits being completed less frequently, while the remaining CMH services were primarily virtual with clients having limited in-person access to the CMH facility for more than 1 year. This CMH setting also represents a public mental health system encompassing the diversity of the midwestern United States (race, ethnicity, socioeconomic status, organizational services, insurance/payment types, etc.). Currently the site is serving over 700 clients with schizophrenia spectrum disorders and this region of the United States has a rate of 14.3 deaths per 100,000 total population suicide, which is a 33% increase since 1999 [27].

Providers were given a link to view and sign a written consent form via Research Electronic Data Capture (REDCap) software and subsequently responded to qualitative questions related to the COVID-19 pandemic (described in the measurement section below). Recruitment and data collection continued until saturation was reached [28]. All data were collected between November and December of 2020 and Institutional Review Board approval was obtained.

### Measurement

**Fig. 1:**
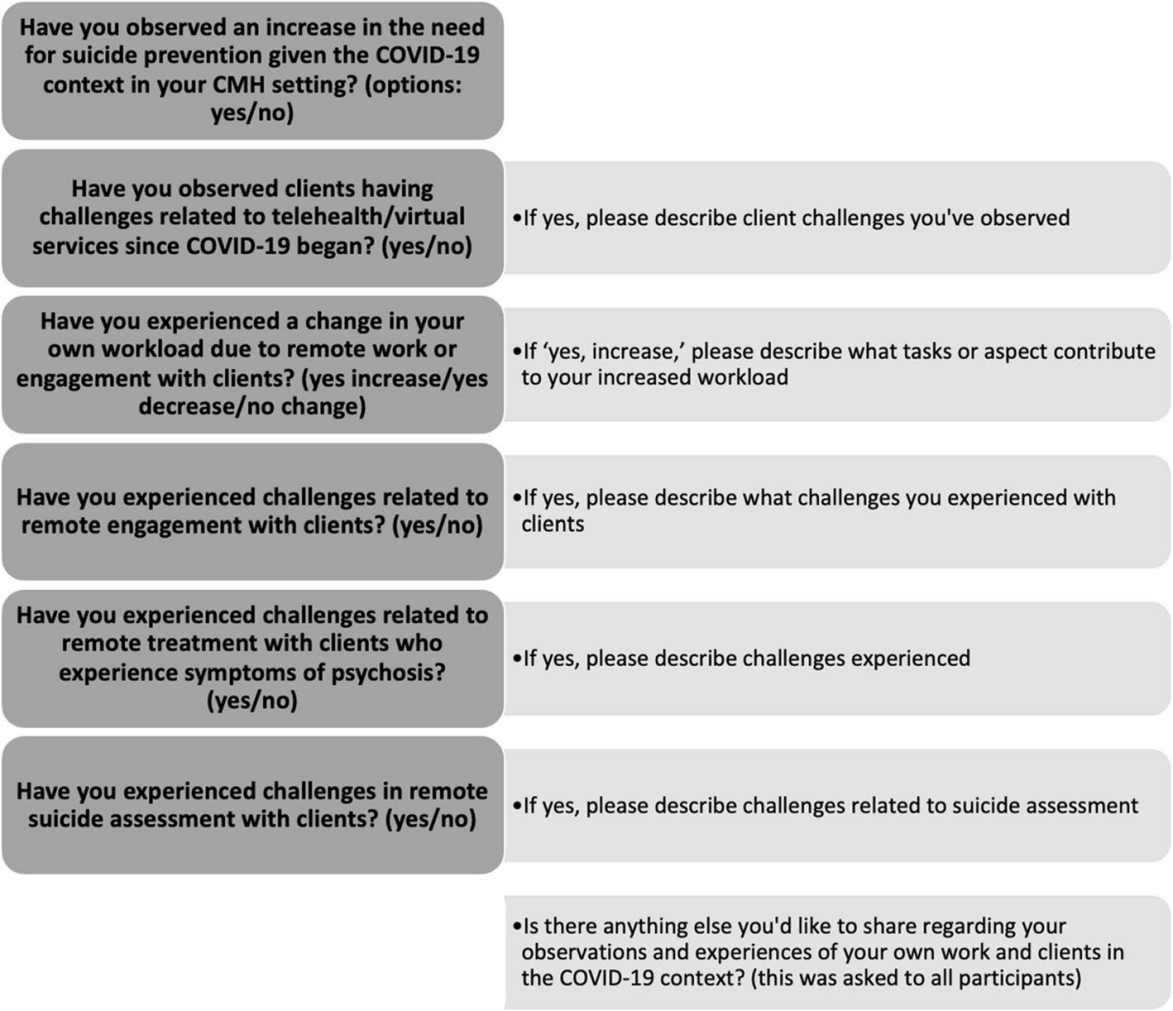
Questions given to providers

Qualitative COVID-19-related questions were preceded by introductory questions about demographic characteristics (e.g., provider age and gender) and practice-related characteristics (e.g., provider license type and duration of practice experience) to increase understandings of the sample. Data collection involved approximately 30 min of time for providers. Prior to question design, it was apparent in conversations with staff and leadership in the CMH setting that many challenges were being faced by clients and providers, thus, questions were focused on challenges with the goal of gaining understandings of potential barriers to service delivery to develop potential solutions and strategies for challenge mitigation. A total of 5 qualitative questions were established by the investigative team, with prior experience in developing qualitative questions, and sought to explore the following within the COVID-19 pandemic-context: 1) provider observations of challenges related to telehealth and virtual services, 2) provider workload changes, 3) challenges experienced in remote engagements with clients, 4) challenges related to engagement with clients who experience symptoms of psychosis, and 5) challenges related to remote suicide assessment. As shown in Fig. 1, qualitative questions were preceded by introductory yes/no questions related to challenges being present, and an additional qualitative question was asked at the end of the survey to gather any additional information, experiences, or observations.

### Data Analysis

Demographic and practice-related data were viewed using SPSS27 (e.g., number and percentage for categorical information, means and standard deviations for continuous information) and qualitative question data analyzed using Dedoose. We first viewed demographic and practice-related data to better understand the sample, followed by qualitative question data analysis with an aim of expanding on descriptive details of the sample (Creswell et al., 2017) [29]. For open-ended qualitative questions, responses were independently coded by two Master’s-level research assistants (JLV and JH) in preparation for codebook development. An open coding technique was used to generate themes across the 6 qualitative questions (Saldana, 2016) [30] and grounded theory methods were utilized for analysis [31]. After an initial round of coding, the lead author (LAB) met with both research assistants to discuss emerging themes from the qualitative data, reviewed field notes from the coding process, and agreed upon a codebook. The research assistants conducted a second round of coding with use of the established codebook and the lead author resolved any disagreements to achieve inter-coder consistency. Themes were ultimately organized into a final framework and findings are presented in the results section below. Strategies for qualitative rigor [32] in the current study include: 1) analytic triangulation (i.e., more than one qualitative coder was involved), 2) audit trail, and 3) member checking.

## Results

Demographic and practice-related provider data are presented in Table 1. Providers (*n* = 12) were on average 35.67 years of age (SD = 6.387), most often identified as female (*n* = 8, 66.7%), and all (*n* = 12) identified as White and non-Hispanic/Latinx. As is common in CMH settings in the US, all providers identified as social workers with 11 having a master’s degree in social work and 1 reporting being currently enrolled in a social work master’s degree program. Providers either had a social work license (LMSW or LCSW; *n* = 9, 75%), limited social work license (LLMSW; *n* = 2, 16.67%), or were a Master of Social Work Student in training (MSW student; *n* = 1. 8.33%). All providers (*n* = 12) endorsed having direct contact with clients with the majority in a mental health therapist, clinician, or case manager role (*n* = 10, 83.3%). The average duration of work experience providing services in the mental health field was 5 years and 10 months (SD = 4 years and 1 month) with a range from 6 months to 14 years. The average duration of work experience in their current CMH setting was 4 years and 6 months (SD = 3 years and 1 month).

**Table 1:** Demographic and practice-related provider data (*n* = 12)

Characteristics and Questions	*N*	%
Age (M ± SD)	12	35.67 ± 6.387
25–29	4	33.33
30–34	4	33.33
35–39	2	16.67
40–45	2	16.67
Gender	12	
Female	8	66.67
Male	4	33.33
Race		
American Indian or Alaska Native	0	0
Asian	0	0
Black or African American	0	0
Native Hawaiian/Pacific Islander	0	0
White	12	100
Other	0	0
Ethnicity		
Non-Hispanic/Latinx	12	100
Hispanic/Latinx	0	0
Licensing Status		
Social Work License (LMSW/LCSW)	9	75
Limited Social Work License (LLMSW)	2	16.67
Social Work (MSW) Student	1	8.33
Role in Community Mental Health (CMH)		
Clinician (Therapist/Clinician/Case Manager)	10	83.3
Clinical Supervisor	1	8.33
Administrator	1	8.33
Years in Mental Health Field (M ± SD)		5.83 ± 4.07
0–4.99	6	50
5–9.99	3	25
10–14	3	25
Years at CMH (M ± SD)		4.49 ± 3.04
0–2.99	4	33.33
3–5.99	4	33.33
6 +	4	33.33
Have you observed an increase in the need for suicide prevention given the COVID-19 context in your CMH setting?		
Yes	8	66.7
No	4	33.3
Have you observed clients having challenges related to telehealth/virtual services since COVID-19 began?		
Yes	8	66.7
No	4	33.3
Have you experienced a change in your own workload due to remote work or engagement with clients?		
Yes, increase in workload	7	58.3
Yes, decrease in workload	1	8.3
No change	4	33.3
Have you experienced challenges related to remote engagement with clients?		
Yes^a^	7	58.3
No	2	16.7
N/A not a clinician or not doing remote engagement with clients	3	25.0
Have you experienced challenges related to remote treatment with clients who experience symptoms of psychosis?		
Yes^a^	8	66.7
No	1	8.3
N/A not a clinician or not doing remote treatment with clients who experience symptoms of psychosis	3	25.0
Have you experienced challenges in remote suicide assessment with clients?		
Yes^b^	4	33.3
No	3	25.0
N/A not a clinician or not doing remote suicide assessment with clients	5	41.7

^a^Among the 9 providers who delivered remote services with clients, 77.8% experienced challenges in remote engagement and 88.9% in remote treatment of psychosis

^b^Among the 7 providers wo engaged in remote suicide assessment with clients, 57% experienced challenges in remote assessment of suicide

### Provider Observations of Client Challenges

All qualitative data findings are presented in Table 2. The majority of providers (66.7%, *n* = 8) observed clients having challenges related to diminished access to telehealth and virtual mental health services during the COVID-19 pandemic. Challenges observed by providers resulted in 3 themes: 1) logistic challenges (qualitative responses: limited availability of a device capable for video and internet, government-supported phones do not always accept restricted calls from providers who use a blocked number), 2) engagement challenges (less ability to engage in individual and group therapy, less comfort engaging with providers remotely, lower therapeutic rapport), and 3) clinical challenges (more agitation, more physical altercations, heightened anxiety, and increased social isolation). Providers shared the following:

*“Clients have limited access to devices capable of all telehealth modalities. Also, doc [doctor] phone numbers come through restricted numbers and some government-supported phones do not accept restricted phone numbers, so when docs are working remotely, certain clients cannot be contacted for even a phone visit by a prescriber.” -provider 1*

*“I’ve seen more agitation in general with clients, specifically during the actual ‘stay home’ period of the pandemic. I had incidents of physical altercations? between residents in homes where there had been little or no signs of aggression in several years.” -provider 3*

*“Prior to the pandemic, our clients were socially isolated due to the nature of severe mental illness. Now they are even more so now as they are not able to attend psychosocial activities or group therapy which has led to worsening of symptoms.” -provider 7*

**Table 2:** Themes of provider responses to qualitative question topics

Qualitative Topic	Theme	Description
Provider observations of client challenges	1. Logistic challenges	Limited availability of a device capable for video and internet, government-supported phones don’t always accept restricted calls from providers who use a blocked number
	2. Engagement challenges	Less ability to engage in individual and group therapy, less comfort engaging with providers remotely, less therapeutic rapport
	3. Clinical challenges	More client agitation, physical altercations, social isolation, anxiety
Provider workload changes	1. Logistic challenges	Assisting clients with technology and troubleshooting challenges, capturing signatures from clients on remote documentation, separating services previously grouped together
	2. Service need	Greater caseload due to increased need for services
	3. Staffing challenges	Providers not passing COVID-19 screening questionnaire, needing to quarantine, becoming ill, and taking sick days to manage mental health and stress
	4. New tasks	Assessing for physical health symptoms
Provider challenges in remote client engagement	1. Logistic challenges	Clients have limited access to technology and devices for telehealth, clients may not respond to phone or phone is not on, clients are less likely to reach out because it is more difficult remotely
	2. Health concerns	Clients concerned about COVID-19 and don’t want to go in for in person services
	3. Service delivery challenges	Harder to engage/build rapport with clients remotely, some services are not possible to deliver remotely, difficult to assess mental status, less accurate information provided by clients
Provider challenges in remote treatment of psychosis	1. Assessment challenges	Abnormal Involuntary Movement Scale (AIMS) via telehealth is very challenging, responses to internal stimuli are hard to assess for remotely, mental status exam is hard to administer remotely, Activities of Daily Living (ADL) are hard to assess for remotely
	2. Engagement challenges	Psychosis makes it difficult to engage in telehealth, paranoia may limit desire to engage, social isolation is common among individuals with psychosis and is the pandemic has worsened
	3. Service delivery challenges	Some services are not possible to deliver remotely
Provider challenges in remote suicide assessment	1. Assessment challenges	Difficult to conduct mental status exam, hard to assess without seeing facial expressions and/or body movements, not as possible to gauge affect, easier for clients to evade questions
	2. Rapport challenges	Hard to build report well and find relatable environmental factors

### Provider Workload Changes

Slightly more than half of the 12 providers (58.3%, *n* = 7) noted their workload increased within the context of the pandemic. Of those who noted an increase, the following 4 themes emerged pertaining to tasks or aspects of service delivery were negatively impacting workload: 1) logistic challenges (assisting clients with telehealth, capturing signatures from clients on documentation, separating services previously grouped together), 2) service need (greater caseload due to increased need for services), 3) staffing challenges (providers not passing COVID-19 screening questionnaire, needing to quarantine, becoming ill, and taking sick days to manage mental health and stress), and 4) new tasks (assessing more for physical health symptoms). Providers shared the following:

*“Many people have been off due to quarantining or taking sick days off for mental health to deal with the stress of COVID-19's repercussions. This understaffing leads to a cycle of workers having to see more clients daily, spending less time with each client, having less time available for paperwork and more paperwork, and causes more stress to the workers/interns. Since this makes it so less time is spent with each client, it increases the risk for crisis situations which also creates more stress/work for the team.” -provider 2*

*“We are having to assess for additional health concerns because of COVID-19 and have had to separate services that are typically grouped together. Such as, medication reviews and injections, and shopping groups with multiple consumers.” -provider 9*

*“In most circumstances, clients are needing telehealth and virtual services to facilitate medication reviews, and many clients don’t have phones or devices for telehealth, which means I assist with facilitation of telehealth services by seeing them with a tablet so they can attend the virtual medication review.” -provider 12*

### Provider Challenges in Remote Client Engagement

Of all participants, 7 (58.3%) endorsed challenges related to telehealth/remote engagement with clients. Importantly, only 9 of the 12 participants provided remote services to clients, therefore among the 9 who engaged remotely, a total of 7 experienced challenges (77.8%). Challenges experienced by providers resulted in 3 themes: 1) logistic challenges (clients have limited to technology and devices for telehealth, clients don’t respond to phone or phone is not on, clients are less likely to reach out because it is more difficult remotely), 2) health concerns (clients concerned about COVID-19 and do not want to go in for in person services), and 3) service delivery challenges (harder for providers to engage/build rapport with clients remotely, some services are not possible to deliver remotely, accuracy of information provided by client in assessment). Providers shared the following:

*“Clients often do not have phones, do not reliably answer their phones, or their phone service is shut off. Also, clients rarely have access to video capable devices for telehealth.” -provider 4*

*“Many of our clients who state virtually that they’re doing well were not actually doing well so the lack of eyes through engagement is impactful.” -provider 8*

### Provider Challenges in Remote Treatment of Psychosis

Of the 9 providers who remotely provided services to clients during the pandemic, 88.9% (*n* = 8) endorsed challenges with remote treatment among clients who experience symptoms of psychosis. Challenges experienced by providers resulted in 3 themes: 1) assessment challenges (administering the Abnormal Involuntary Movement Scale [AIMS] via telehealth is very challenging, responses to internal stimuli are hard to assess for remotely, the mental status exam [MSE] is hard to administer remotely, Activities of Daily Living (ADLs) are hard to assess for remotely), 2) symptomatology (psychosis symptoms make it difficult to engage in telehealth, paranoia may limit desire to engage), and 3) service delivery challenges (some services are not possible to deliver remotely). Providers shared the following:

*“If they are not in person, it is hard to assess all the aspects of a mental status exam…or to assess for ADLs when using a phone or tablet.” -provider 5*

*“It’s more challenging for clients experiencing internal stimuli to maintain engagement generally. Being remote limits the ability to redirect or re-engage when clients are experiencing symptoms.” -provider 10*

*“It is difficult for providers to get a sense of symptomatology through telehealth services. Paranoia may limit desire to engage.” -provider 12*

### Provider Challenges in Remote Suicide Assessment

Of the 9 providers who remotely provided services to clients during the pandemic, 7 endorsed engaging in remote suicide assessments with clients, and of whom, 4 (57%) experienced challenges in conducting remote suicide assessments with clients during the pandemic. Challenges resulted in 2 themes: 1) assessment challenges (difficult to conduct mental status exam, hard to assess without seeing facial expressions and/or body movements, easier for clients to evade questions) and 2) rapport challenges (hard to build report well and find relatable environmental factors). Providers shared the following:

*“Over the phone it has been harder to assess clients’ symptomology without seeing body and facial expressions.” -provider 2*

*“Clients may be more likely not to share their thoughts or feelings when there isn’t face-to-face contact.” -provider 5*

*“It is easier for people to dodge your questions on the phone, you can’t build rapport as well, find relatable environmental factors, and it is hard to get to linking and information sharing.” -provider 11*

## Discussion

Literature of the evolving COVID-19 pandemic highlight an impact on mental health service need, utilization, and delivery (Ardebili et. al, 2020; [25, 10, 19], Vizeh et al., 2020). Given the prediction that COVID-19 may disproportionately impact individuals with psychosis [16], a population that is at greater risk for suicide in comparison to the general population (Adyin et al., 2019,Bornheimer, 2020) [14], it is critical that mental health providers can effectively engage, conduct suicide-risk assessments, and deliver services to individuals with psychosis. As a result, greater understandings of the COVID-19 impact on services and delivery are needed from the perspective of providers in CMH with specific attention to challenges in assessing and treating psychosis and suicide risk. Data of the current study indicate CMH providers observed a greater need for suicide prevention, clients facing challenges with telehealth and virtual services, an increase in workload, and challenges with remote engagement, treatment of psychosis, and suicide assessment since the COVID-19 pandemic began. Provider responses to qualitative questions further underscore and expand upon the logistic, engagement, and clinical challenges emerging within the pandemic context.

Logistic challenges emerged as a theme including provider observations of clients facing technology barriers in remote service use, clients reaching out less to providers, some services not being offered remotely due to the need for technology, and provider challenges in engaging with clients due to limited technology. These technology challenges further reinforce the disparaging impact of COVID-19 with technology access for remote engagement with mental health services presenting an inequality gap [33]. While many may have access to smart phones, computers, and reliable internet connections, clients engaging in community-based mental health services often reside in low-income, underserved, or rural areas with limited access to technology that is now needed for virtual services [22]. Some providers shared that they tried to increase access by allowing clients to use their device to meet virtually with psychiatrists and primary care providers. Though providing a device for clients as a method of problem-solving aligns with social work values of compassion, justice, and beneficence, this is also unlikely a standard practice due to resources (e.g., availability of technology and cost) and provider time (e.g., increased caseloads due to greater service needs and staffing challenges). Beaunoyer and colleagues (2020) propose a multi-layer strategy to mitigate digital inequalities involving government, organizations, corporations, and communities. It is suggested that offering alternatives to technology (e.g., allowing a phone call visit instead of a teleconference visit which requires a camera and internet), increasing coverage and capabilities of networks, public funding for increased network access, and donating technology devices to low-income households may increase access to technology. It is also recommended to increase digital literacy with household, family, and community support for device use, tutorials and trainings, and adding digital literacy to public school curriculums.

Limited technology access also serves as a foundation for the rapport and engagement challenges that providers noted. This engagement theme includes provider observations of clients engaging less in services, clients facing challenges in remote engagement due to psychosis symptoms, and provider challenges in remotely building rapport with clients. Findings align with recent literature client and provider engagement challenges since the COVID-19 pandemic began due to technology and service access [25, 34]. These engagement challenges are likely influenced and exacerbated by increased levels of social isolation during the pandemic, already an extant problem for individuals experiencing negative symptoms within psychosis (e.g., reduction in emotional experience and loss of volition; [35]. It is also possible these challenges are influenced by variations in digital literacy, and service delivery organizations may benefit from offering training to their clients in how to engage with providers via smartphones and computers as they navigate online service system portals, audio and video telehealth sessions, setting up calendar reminders for virtual appointments, and more [36]. If the technology gap can be improved by increasing access to remote services (e.g., offering both telephone and videoconferencing options for care) and training for clients to engage in remote services, providers could integrate greater opportunities for individual and group virtual engagement to foster a sense of community and belonging.

In addition to logistic and engagement themes, findings also revealed clinical and service delivery challenges. Providers described clients having increased agitation and anxiety, provider challenges with remote assessment, new tasks of assessing for COVID-related symptoms, and increased caseloads due to greater mental health needs in the community and provider staffing challenges. Assessment was a common theme in which providers shared difficulties in conducting mental status exams in relation to suicide risk and symptoms of psychosis, administering the Abnormal Involuntary Movement Scale (AIMS), and assessing for Activities of Daily Living (ADLs). For example, providers described challenges in assessing affect without seeing facial expressions, client experience and response to internal stimuli, and overall noted greater question evasion by clients. The challenges of assessing a client without being able to observe them are also echoed in recent literature [24], and for those treating psychosis, observation of symptoms is an essential component [37]. Related to service delivery, it was apparent that provider shortage was common due COVID-19 symptoms or illness, quarantine, and using sick time to manage stress and mental health,all of which increased workloads and caseloads.

Beyond troubleshooting technology barriers, findings point towards the need for support and training among providers who have been navigating challenges and practice unknowns in CMH settings and beyond. The COVID-19 pandemic arrived in 2020 with overwhelming demands for mental health providers and healthcare workers overall, raising concerns of trauma exposure, stress, and mental illness (i.e., depression, anxiety, and post-traumatic stress disorder) among providers delivering services [38]. In the current study, providers shared that workload changes were challenging to manage and given it may not be feasible to hire more staff, strategizing for how to manage increased caseloads with bolstered support for providers to prevent burnout is essential. Burnout prevention approaches often involve self-care (e.g., sleep, breaks from work, movement), stress management (e.g., mindfulness, exercise), emotional support and professional mental health treatment [39]. Such approaches can be integrated into service delivery settings like CMH, with the potential to improve provider support and quality of life [40]. In addition to provider-focused strategies to prevent burnout, it is also imperative for agencies and organizations to foster supportive environments for staff. For example, establishing health and safety protocols with monitoring for mental health, efforts to de-stigmatize provider mental health, collaborating with providers and staff on what is needed to improve working conditions, and mindfulness of the disproportionate impact of COVID-19 on poverty-impacted individuals and racial and ethnic minority groups [39]. For specific focus on mental health and trauma, Psychological First Aid for mental health providers may be beneficial to implement in CMH settings as suggested from prior pandemic-related studies (e.g., SARS) focused on healthcare workers [41].

Though providers undergo training in educational programs, during licensure obtainment, and continuing education, most focus on in-person engagement and service delivery as opposed to telehealth and virtual forms of service delivery. An international survey (*n* = 1206) across 100 countries revealed that approximately 49.1% of clinicians reported that they had not received any training in teletherapy (i.e., telephone and videoconference; [42]. In the United States, studies prior to the COVID-19 pandemic show approximately 25% of clinicians used telehealth with a lack of available training being cited as a barrier [43, 44]. Therefore, it is essential for trainings to be established and implemented for providers to gain skills and confidence in remote engagement, assessment, and service delivery including the nuances of mental status and emotion expression. Assessing for symptoms of psychosis and suicide risk, particularly of focus in the current study, are challenging to do remotely (e.g., AIMS) and likely require additional clinical training and skills. In addition to the shift towards virtual service delivery with clients, many providers have also navigated a shift to virtual supervision [39]. Therefore, approaches to practice and supervision must be adjusted with space for real-time processing of changes and subsequent modifications as needed. Overall, new and additional training for clinicians both in the context of continuing education and also provided at service delivery sites like CMH may alleviate some of the clinical challenges faced resulting from telehealth and virtual service delivery.

### Limitations

First, the findings emerged from a small sample of providers in a CMH setting of a northern midwestern region of the United States. Thus, social work provider perspectives in the sample may differ from providers of other disciplines, other mental health settings, and geographical areas across the world. It is essential for future investigations to examine a range of experiences and impacts of the COVID-19 pandemic among mental health providers across characteristics, contexts, and settings. Second, providers shared observations of client challenges within the pandemic context who were reportedly engaged in CMH services, therefore emerging themes of pandemic-related challenges may differ for clients who are not engaged with services. Third, providers most often identified as female, White, and non-Hispanic/Latinx, thus the sample is not representative of all providers across the United States and the globe. Fourth, although themes did not emerge in the data about positive changes or outcomes in relation to the pandemic, our interview did not specifically ask or probe for positive aspects of the pandemic context. We did, however, have an open-ended question asking for any additional observations or experiences at the end of the interview, with no positive responses emerging. It is important to be mindful that while many challenges emerged, it is also very possible that there are benefits to the pandemic context and virtual service delivery for providers (e.g., some people may enjoy working remotely from home or not having to drive to work). Lastly, data were qualitative, cross-sectional in nature, and collected between November and December of 2020. As a result, statistical investigations of findings did not occur and fluctuations or patterns of provider experiences were not examined across various points of time and waves of the COVID-19 pandemic.

## Conclusions

Despite growing literature of the COVID-19 pandemic’s impact on mental health, less is known about changes in mental health services and delivery from the perspective of mental health providers and related to service delivery among individuals with psychosis and risk for suicide. Study findings highlight the logistic, engagement, and clinical challenges of clients and providers in a CMH setting. Provider perspectives are essential to inform efforts to build resources and problem-solve challenges and barriers with an overall goal of improving access, feasibility, and quality of mental health service delivery in CMH settings and beyond. The recommendations informed by the study’s findings are not only vital for the unfolding and evolving COVID-19 pandemic, but also in preparation for ongoing shifts in service delivery as technology evolves. 

## Data Availability

Data will be made available through the NIH Data Archive (NDA) upon completion of the study.

## Abbreviations

ACT: Assertive Community Treatment
CMH: Community Mental Health
NIMH: National Institute of Mental Health
CDC: Center for Disease Control
MSW: Master of Social Work
LMSW: Licensed Master of Social Work
LCSW: Licensed Clinical Social Worker
SD: Standard Deviation
AIMS: Abnormal Involuntary Movement Scale
ADL: Activity of Daily Living

## References

[1] Cole CL, Waterman S, Stott J, Saunders R, Buckman JEJ, Pilling S, Wheatley J. Adapting IAPT services to support frontline NHS staff during the Covid-19 pandemic: the Homerton Covid Psychological Support (HCPS) pathway. Cogn Behav Ther. 2020;13:e12. 10.1017/S1754470X20000148 PMC7235312 32454891

[2] Ivbijaro G, Brooks C, Kolkiewicz L, Sunkel C, Long A (2020). Psychological impact and psychosocial consequences of the COVID 19 pandemic: Resilience, mental well-being, and the coronavirus pandemic. Indian J. Psychiatry.

[3] Rahul P, Chander KR, Murugesan M, Anjappa AA, Parthasarathy R, Manjunatha N, Math SB (2021). Accredited Social Health Activist (ASHA) and her role in district mental health program: Learnings from the COVID 19 pandemic. Community Ment Health J.

[4] Kontoangelos K, Economou M, Papageorgiou C (2020). Mental health effects of COVID-19 pandemia: a review of clinical and psychological traits. Psychiatry Investig.

[5] Duan L, Zhu G (2020). Psychological interventions for people affected by the COVID-19 epidemic. Lancet Psychiat.

[6] Sanderson WC, Arunagiri V, Funk AP, Ginsburg KL, Krychiw JK, Limowski AR, Stout Z (2020). The nature and treatment of pandemic-related psychological distress. J Contemp Psychother.

[7] Bao Y, Sun Y, Meng S, Shi J, Lu L (2020). 2019-nCoV epidemic: Address mental health care to empower society. The Lancet.

[8] Ferrando SJ, Klepacz L, Lynch S, Shahar S, Dornbush R, Smiley A, Bartell A (2020). Psychiatric emergencies during the height of the COVID-19 pandemic in the suburban New York City area. J Psychiatr Res.

[9] Xiang YT, Yang Y, Li W, Zhang L, Zhang Q, Cheung T, Ng CH (2020). Timely mental health care for the 2019 novel coronavirus outbreak is urgently needed. Lancet Psychiat.

[10] Reading Turchioe M, Grossman LV, Myers AC, Pathak J, Creber RM (2021). Correlates of Mental Health Symptoms Among US Adults During COVID-19, March–April 2020. Public Health Rep.

[11] Thakur V, Jain A. COVID 2019-suicides: A global psychological pandemic. Brain Behav Immun. 2020;88:952-953. 10.1016/j.bbi.2020.04.062 PMC7177120 32335196

[12] Gunnell D, Appleby L, Arensman E, Hawton K, John A, Kapur N, Yip PS (2020). Suicide risk and prevention during the COVID-19 pandemic. Lancet Psychiat.

[13] Aydin M, Ilhan BC, Tekdemir R, Çokünlü Y, Erbasan V, Altınbaş K (2019). Suicide attempts and related factors in schizophrenia patients. Saudi Med J.

[14] Bornheimer LA, Zhang A, Li J, Hiller M, Tarrier N (2020). Effectiveness of suicide-focused psychosocial interventions in psychosis: a systematic review and meta-analysis. Psychiatr Serv.

[15] Jacobs DG, Baldessarini RJ, Conwell Y, Fawcett JA, Horton L, Meltzer H, ... & Simon RI. Assessment and treatment of patients with suicidal behaviors. APA Practice Guidelines. 2010:1–183.

[16] Anglin DM, Galea S, Bachman P (2020). Going upstream to advance psychosis prevention and improve public health. JAMA Psychiat.

[17] Brown E, Gray R, Monaco SL, O'Donoghue B, Nelson B, Thompson A, ... & McGorry P. The potential impact of COVID-19 on psychosis: a rapid review of contemporary epidemic and pandemic research. Schizophr Res. 2020;222:79-87. 10.1016/j.schres.2020.05.005 PMC7200363 32389615

[18] Adamou M (2005). Community service models for schizophrenia: evidence-based implications and future directions. Psychiatry (Edgmont).

[19] Lai J, Ma S, Wang Y, Cai Z, Hu J, Wei N, Hu S (2020). Factors associated with mental health outcomes among health care workers exposed to coronavirus disease 2019. JAMA Netw Open.

[20] Vizheh M, Qorbani M, Arzaghi SM, Muhidin S, Javanmard Z, Esmaeili M. The mental health of healthcare workers in the COVID-19 pandemic: A systematic review. J Diabetes Metab Disord. 2020;19(2):1–12. 10.1007/s40200-020-00643-9 PMC7586202 33134211

[21] Pierce, B. S., Perrin, P. B., Tyler, C. M., McKee, G. B., & Watson, J. D. (2021). The COVID-19 telepsychology revolution: A national study of pandemic-based changes in U.S. mental health care delivery. *American Psychologist, 76*(1), 14–25. 10.1037/amp0000722 32816503

[22] Weigel G, Ramaswamy A, Sobel L, Salganicoff A, Cubanski J, Freed M. Opportunities and barriers for telemedicine in the US during the COVID-19 emergency and beyond. Women’s Health Policy. 2020. Accessed 1 Mar 2022. https://www.kff.org/womens-health-policy/issue-brief/opportunities-and-barriers-for-telemedicine-in-the-u-s-during-the-covid-19-emergency-and-beyond/.

[23] Buckman JE, Saunders R, Leibowitz J, Minton R (2021). The barriers, benefits and training needs of clinicians delivering psychological therapy via video. Behav Cogn Psychother.

[24] Uscher-Pines L, Sousa J, Raja P, Mehrotra A, Barnett ML, Huskamp HA (2020). Suddenly becoming a “virtual doctor”: Experiences of psychiatrists transitioning to telemedicine during the COVID-19 pandemic. Psychiatr Serv.

[25] Ardebili M, Naserbakht M, Bernstein C, Alazmani-Noodeh F, Hakimi H, Ranjbar H (2020). Healthcare providers experience of working during the COVID-19 pandemic: A qualitative study. Am J Infect Control.

[26] Tong A, Sainsbury P, Craig J (2007). Consolidated criteria for reporting qualitative research (COREQ): a 32-item checklist for interviews and focus groups. Int J Qual Health Care.

[27] CDC. (2019). Suicide Mortality by State: Michigan. Accessed May 1, 2021. https://www.cdc.gov/nchs/pressroom/sosmap/suicide-mortality/suicide.htm.

[28] Saunders B, Sim J, Kingstone T, Baker S, Waterfield J, Bartlam B, Jinks C (2018). Saturation in qualitative research: exploring its conceptualization and operationalization. Qual Quant.

[29] Creswell JW, Clark VLP. Designing and conducting mixed methods research. Thousand Oaks, CA: Sage publications; 2017.

[30] Saldaña J. The coding manual for qualitative researchers. Thousand Oaks, CA: Sage publications; 2021.

[31] Charmaz K. Constructing grounded theory. Thousand Oaks, CA: Sage publications; 2014.

[32] Padgett DK. Qualitative methods in social work research (Vol. 36). Thousand Oaks, CA: Sage publications; 2016.

[33] Beaunoyer E, Dupéré S, Guitton MJ (2020). COVID-19 and digital inequalities: Reciprocal impacts and mitigation strategies. Comput Hum Behav.

[34] Cadel L, Marcinow M, Sandercock J, Dowedoff P, Guilcher SJ, Maybee A, Kuluski K (2021). A scoping review of patient engagement activities during COVID-19: More consultation, less partnership. PLoS ONE.

[35] Lysaker PH, Shea AM, Buck KD, Dimaggio G, Nicolò G, Procacci M, Rand KL (2010). Metacognition as a mediator of the effects of impairments in neurocognition on social function in schizophrenia spectrum disorders. Acta Psychiatrica Scandanavia.

[36] Hoffman L, Wisniewski H, Hays R, Henson P, Vaidyam A, Hendel V, Torous J (2020). Digital opportunities for outcomes in recovery services (DOORS): A pragmatic hands-on group approach toward increasing digital health and smartphone competencies, autonomy, relatedness, and alliance for those with serious mental illness. J Psychiatr Pract.

[37] Sharp IR, Kobak KA, Osman DA (2011). The use of videoconferencing with patients with psychosis: a review of the literature. Ann Gen Psychiatry.

[38] Søvold LE, Naslund JA, Kousoulis AA, Saxena S, Qoronfleh MW, Grobler C, Münter L. Prioritizing the mental health and well-being of healthcare workers: an urgent global public health priority. Front Public Health. 2021;9:1-12. 10.3389/fpubh.2021.679397 PMC8137852 34026720

[39] Gruber J, Prinstein MJ, Clark LA, Rottenberg J, Abramowitz JS, Albano AM, ... & Weinstock LM. Mental health and clinical psychological science in the time of COVID-19: Challenges, opportunities, and a call to action. Am Psychol. 2020;76(3):409-426. 10.1037/amp0000707 PMC7873160 32772538

[40] Dyrbye LN, Shanafelt TD, Sinsky CA, Cipriano PF, Bhatt J, Ommaya A, West CP, et al. Burnout among health care professionals: A call to explore and address this underrecognized threat to safe, high-quality care. NAM Perspectives. Discussion Paper. Washington DC: National Academy of Medicine; 2017.

[41] Maunder RG, Peladeau N, Leszcz M, Romano D, Savage D, Rose M, Reg O, et al. Applying the Lessons of SARS to Pandemic Influenza An Evidence-based Approach to Mitigating the Stress Experienced by Healthcare Workers. Can J Public Health. 2008;99(6):486-88. 10.1007/BF03403782 PMC5148615 19149392

[42] Montoya MI, Kogan CS, Rebello TJ, Sadowska K, Garcia-Pacheco JA, Khoury B, Reed GM (2022). An international survey examining the impact of the COVID-19 pandemic on telehealth use among mental health professionals. J Psychiatr Res.

[43] Pierce BS, Perrin PB, McDonald SD (2020). Demographic, organizational, and clinical practice predictors of US psychologists’ use of telepsychology. Prof Psychol Res Pract.

[44] Perle JG, Burt J, Higgins WJ (2014). Psychologist and physician interest in telehealth training and referral for mental health services: An exploratory study. J Technol Hum Serv.

